# Genetic diversity and bioinformatic analysis in the L1 gene of HPV genotypes 31, 33, and 58 circulating in women with normal cervical cytology

**DOI:** 10.1186/s13027-023-00499-7

**Published:** 2023-03-23

**Authors:** Mina Mobini Kesheh, Sara Shavandi, Jalil Azami, Maryam Esghaei, Hossein Keyvani

**Affiliations:** 1 Department of Virology, School of Medicine, Iran University of Medical Sciences, Tehran, Iran; 2 Department of Industrial and Environmental Biotechnology, National Institute of Genetic Engineering and Biotechnology, Tehran, Iran; 3 Faculty of Veterinary Medicine, Urmia University, Urmia, Iran

**Keywords:** L1 gene, Human papillomavirus (HPV), Lineage phylogeny, Selective pressure, Homology models

## Abstract

**Background:**

HPV-31, -33, and -58, along with HPV-45 and -52, account for almost 11% of HPV-associated cancers. Our previous studies showed that after HPV-16 and -51, HPV-58 was common and HPV-31 was as frequent as HPV-18 among Iranian women with normal cytology. Hence, in this study, we aimed to investigate the intra-type variations in L1 genes of HPV-58, -31, and -33 to find the predominant lineages circulating in women with normal cytology.

**Methods:**

Complete coding sequencing of the L1 gene was amplified and nucleotide and amino acid sequences were compared to those of the references. The selective pressure on L1 protein and whether the variations of the L1 genes embed in L1 loops, or N-glycosylated sites were also investigated.

**Results:**

B1, A, and A1 (sub)lineages were common in the HPV-58, -33, and -31 samples, respectively. Ninety nucleotide mutations were observed. Twenty nine nucleotide changes corresponded to nonsynonymous substitutions in which seventeen mutations were located in L1 loops. Only one codon position in HPV-58 sequences was found as the positive selection. No difference was observed in N-glycosylation sites between reference and understudied amino acid sequences.

**Conclusion:**

In the current study, we reported, for the first time, the (sub) lineages, amino acid, and genetic diversity in the L1 gene of circulating HPV-58, -33, and -31, in women with normal cytology, in Iran. Such studies can not only have epidemiological values, but also aid to set vaccination programs.

**Supplementary Information:**

The online version contains supplementary material available at 10.1186/s13027-023-00499-7.

## Background

Human papillomaviruses (HPVs) are small, non-enveloped, double stranded DNA viruses with a genome consisting of approximately 8000 bp, which contains of early region (E6, E7, E1, E2, E4, and E5) and late region (L1, L2) [1]. HPVs cause benign lesions (warts) and cancers in humans. Up to now, more than 220 HPVs have been identified and classified into five genera of alpha, beta, gamma, mu, and nu [2], among which 14 HPV genotypes are known as high-risk (HR) HPVs (16, 18, 31, 33, 35, 39, 45, 51, 52, 56, 58, 59, 66, and 68) [3]. HR HPVs are involved in 10% of humans’ cancer. Cervical cancer, which is the third most prevalent cancer among women, results from the persistent infection of HPVs. HPVs are also the main reason for anal cancer as well as other carcinomas in vulva, vagina, and penis [4]. HPV genotypes 31, 33, and 58, along with HPV-45 and 52, account for almost 15% of cervical cancers and 11% of HPV-associated cancers which can be prevented by 9-valent vaccine [3]. This vaccine is not available in developing countries which raises the importance of four HPV genotypes other than HPV-16 and -18, which can be controlled by 2- and 4-valent vaccines.

Considering the fact that HPV types are able to diversify their genetic content from their whole genome, they can also be separated into lineages and sub-lineages, diverging from 1 to 10% and 0.5 to 1%, respectively [5]. There are three lineages in HPV-31: A, B and C; three lineages in HPV-33: A, B and C and two sub-lineages (A1 and A2); four lineages in HPV-58 (A, B, C, and D) with seven sub-lineages (A1, A2, A3, B1, B2, D1, and D2) [6, 7]. With the diversification of lineages in HPV genotypes, the prognosis of HPV infections may change significantly as lineages have different abilities in terms of developing cervical cancer, persistence and escaping the immune system [5]. Indeed, one of the effective immune evasion mechanisms developed by HPVs are mutations occurring, especially, in L1 gene, which hamper the induction of B-cell and T-cell associated immune responses [8]. With the detection of cross reactivity among HPV vaccine recipients in response to non-HPV vaccine genotypes, HPV-16 L1 loops are used as a model to investigate the immunogenic domains in other HR HPVs [9]. The predominant lineage of HPV-45 which was B as reported in our previous study, with the same criterion for sampling [10]. Also, we found B variants as a frequent lineage in HPV-52 samples (unpublished data). Thus, in the current study, we decided to report the prevalent variants of HPV-31, -33, and -58 and investigate the effect of amino acid changes on selective pressure, N-glycosylation sites, and whether these mutations were located in the five external loos; BC, DE, EF, FG, and HI.

## Methods

### Ethics statement

The current study was approved by the ethics committee of Iran University of medical sciences, Tehran, Iran with ID IR.IUMS.REC 1396.32308. According to Helsinki declaration, all patients were informed by a written consent prior to sampling and their information was attentively protected.

### Sample collection

In this cross-sectional study, 32, 21, and 11 Thin Prep pap samples with normal cytology were included that were positive for the single infection of HPV-58, -31, and -33, respectively. Indeed, the samples were collected from women who were referred to hospitals affiliated to Iran University of medical sciences in Tehran province for routine screening tests from October 2019 to September 2021.

### HPV DNA extraction and the L1 gene amplification

HPV DNA was isolated by using QIAamp DNA Mini kit (Qiagen GmbH, Germany). The positive samples were approved by High + Low PapillomaStrip kit (Opegen Kit, Spain) for HPV genotyping, according to the manufacturer’s instructions. The L1 gene in samples were amplified, using 50 µl of total PCR reaction, including 33.9 µl DNase-free water, 5 µl 10 × reaction buffer, 4 µl dNTP mix (10 mM for each), 0.8 µl of each forward and reverse specific primers (10 pmol/µl), 0.5 µl mi-Pfu DNA polymerase (metabion, Germany), and 5 µl DNA template. Also, β-globin primers were used as control and to determine the quality of DNA. The primers and thermocycler programs that used in this study are shown in Table 1.

**Table 1:** The primers and *programs of* the *thermocycler* with temperatures cycle

Primers	5′-3′ sequences	PCR product	PCR conditions	References
HPV-31 (F) HPV-31 (R)	CCTACAACGCCACAAGTGTC CAATACAGCACAAGCACATACAC	1667 bp	Primary denaturation: 95 °C, 120 s Denaturation: 95 °C, 20 s Annealing: 58 °C, 25 s × 43 Extension: 72 °C, 85 s Final extension: 72 °C, 300 s	This study
HPV-33 (F) HPV-33 (R)	ACCATTGTTGTAGACGGTGC GACAAGTACATAGAACATGCACAC	1760 bp	Primary denaturation: 95 °C, 120 s Denaturation: 95 °C, 20 s Annealing: 56 °C, 25 s × 43 Extension: 72 °C, 95 s Final extension: 72 °C, 300 s	This study
HPV-58 (F) HPV-58 (R)	CGTACCAGTAATGTGTCCATACC GCACCTTACTCATAGATACACCC	1950 bp	Primary denaturation: 95 °C, 120 s Denaturation: 95 °C, 20 s Annealing: 60 °C, 25 s × 43 Extension: 72 °C, 105 s Final extension: 72 °C, 300 s	This study

### Phylogenetic trees

Multiple sequence alignment (MSA) of sequenced samples was done and phylogenetic trees were drawn by using maximum likelihood method, Kimura 2-parameter method in the bootstrap test, 1000 replicates, by Mega 10 software [11]. The sequences with Genbank accession numbers were used for each sub-lineage as references.

### N-glycosylation analysis

The understudied L1 gene nucleotides were translated to amino acid sequences through an online tool of the European Bioinformatics Institute (EMBL-EBI, EMBOSS Transeq Tool) (https://www.ebi.ac.uk/Tools/st/emboss_transeq). Complete amino acid sequences of HPV-31, -33, and -58 isolates were compared with the references UniProt accession numbers P17388, P06416, and P26535, respectively, to determine the changes. Consequently, NetNGlyc 1.0 server was applied to predict N-glycosylated sites through artificial neural networks and threshold 0.5 defined by the conserved Asparagine-Xaa-Serine/Threonine sequons [14].

### Selective pressure analysis and homology modeling

Datamonkey was used to detect amino acid sites under selection. This free online server detects selective pressure based on statistical methods, such as the Fixed Effects Likelihood (FEL), in which the rate of synonymous (alpha) and nonsynonymous (beta) substitution is used to estimate the negative and positive selection at each codon based on the neutral model (alpha = beta) and *p* value ≤ 0.1 for a given coding alignment and corresponding phylogeny [12, 13]. The homology models of the HPV genotypes L1 protein were constructed by Swiss-model [14] in comparison with the HPV-16 L1 protein.

## Results

The average age of infected females with HPV-58, -31, and -33 were 33.6, 29.8, and 33, respectively. Two samples from HPV-58 and one sample from HPV-33 were excluded due to the undesirable sequencing, thus we continued the study with 30 and 10 samples for HPV-58 and HPV-33, respectively.

### Phylogenetic trees

**Fig. 1:**
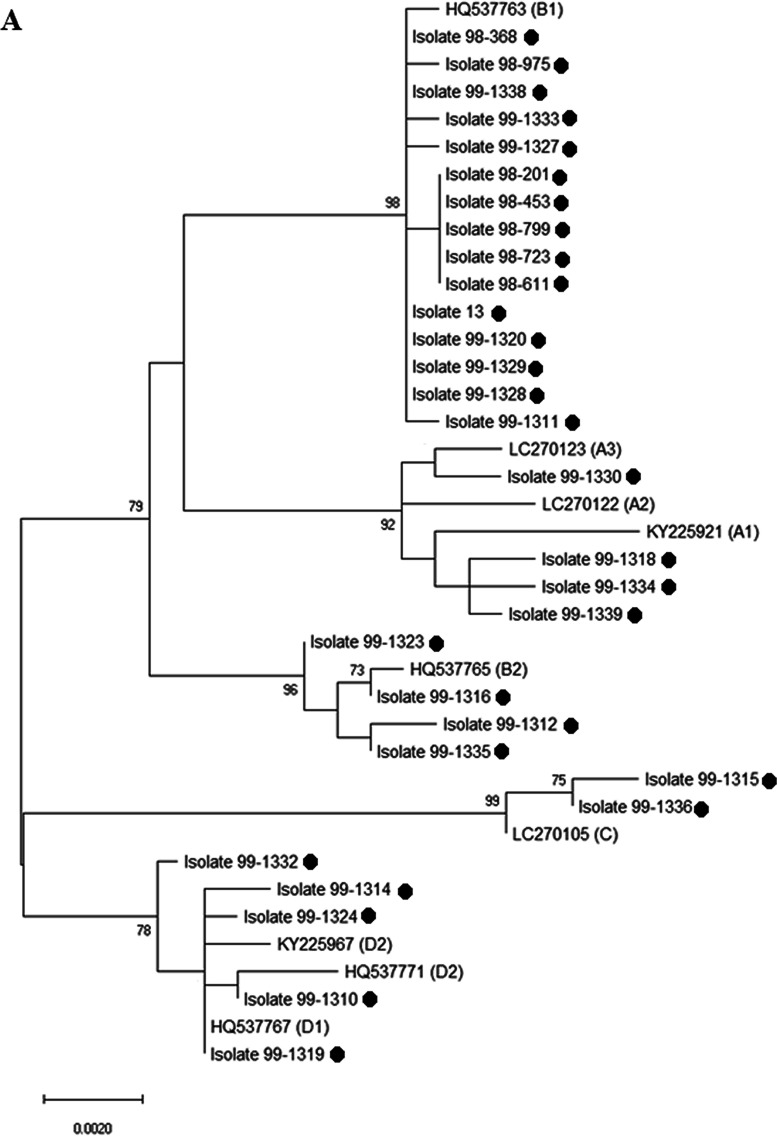

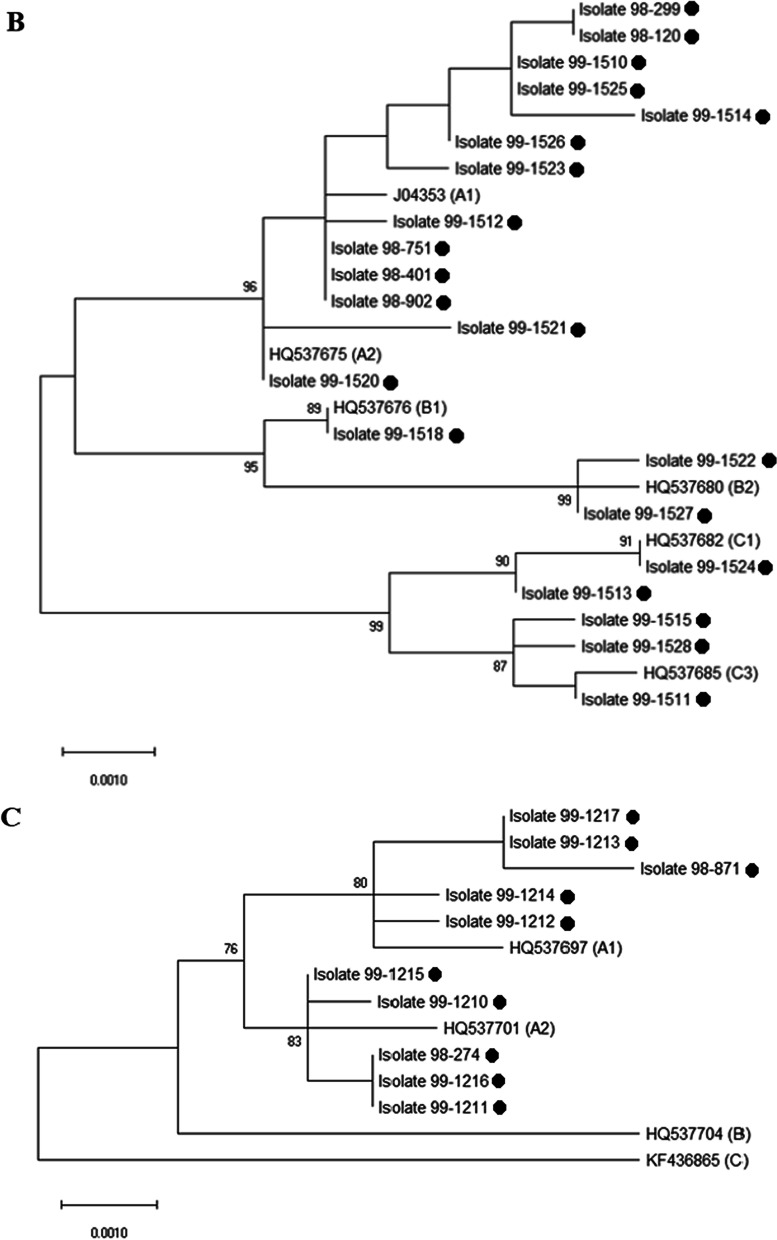
Phylogenetic trees of three HPV genotypes 58 (**A**), 31 (**B**) and 33 (**C**) based on alignments of the L1 genes. The trees were constructed in Mega 10 by using maximum likelihood/Kimura 2-parameter method and 1000 bootstrap replicates and the values greater than 70% are shown above the branches. The isolates from this study are shown with small black circles and remaining accession numbers are available HPV genotypes 31, 33, and 58 sequences in the Genbank as references. All HPV genotypes of this study are available in the NCBI database and Genbank accession numbers OQ412837-82, MT267729, MZ221065-73, and MZ221053-57

The phylogenetic trees were constructed by maximum likelihood method with Mega 10 software (Fig. 1). Four lineages were distinguished in HPV-58, A (13.3%, n = 4), B (63.3%, n = 19), C (6.7%, n = 2), and D (16.7%, n = 5); however, there were six sub-lineages among HPV-58 isolates, including A1 (10.0%, n = 3), A3 (3.3%, n = 1), B1 (50.0%, n = 15), B2 (13.3%. n = 4), D1 (3.3%, n = 1), and D2 (13.4%, n = 4). HPV-31 was divided into three lineages, A (61.9%, n = 13), B (14.3%, n = 3), and C (23.8%, n = 5). Each lineage fell into two sub-lineages, including A1 (57.1%, n = 12), A2 (4.8%, n = 1), B1 (4.8%, n = 1), B2 (9.5%, n = 2), C1 (9.5%, n = 2), and C3 (14.3%, n = 3). All HPV-33 isolates (100.0%, n = 10) accounted for lineage A (50.0% A1 and 50.0% A2).

### Amino acid and genetic variability

#### HPV-31

Nucleotide sequences analysis on the L1 gene of HPV-31 showed 34 changes (Additional file 1: Table S1). The most frequent mutation, which was seen in all isolates, was C1266A, followed by C186T, in 61.9% (n = 13) of isolates. The third most frequent mutations were T370C, C821A, and G1245A, accounting for 38.1% (n = 8) of samples. Eight mutations were found in all lineage C isolates, T30A, A468G, G777A, A799G, T1035G, G1245A, C534T, and C821A; the two formers of which led to T267A and T274N missense mutations, respectively (Table 2).

**Table 2:** Amino acid mutations in all HPV-58, -31, and -33 isolates

HPV genotypes	Positions																			
	Isolate	5	10	144	150	159	163	220	292	296	299	311	325	375	378	383	412	420	422	479
HPV-58	P26535|VL1*	L	A	V	L	S	P	I	K	A	D	V	I	T	G	D	I	D	N	K
	13	–	–	I	F	–	–	–	–	–	–	–	M	–	–	–	V	N	D	–
	98–368	–	–	I	F	–	–	–	–	–	–	–	M	–	–	–	V	N	D	–
	98–201	–	–	I	F	–	–	–	–	–	–	–	M	–	–	–	V	N	D	–
	98–453	–	–	I	F	–	–	–	–	–	–	–	M	–	–	–	V	N	D	–
	98–975	–	–	I	F	–	–	–	–	–	–	–	M	–	–	–	V	N	D	T
	98–799	–	–	I	F	–	–	–	–	–	–	–	M	–	–	–	V	N	D	–
	98–723	–	–	I	F	–	–	–	–	–	–	–	M	–	–	–	V	N	D	–
	98–611	–	–	I	F	–	–	–	–	–	–	–	M	–	–	–	V	N	D	–
	99–1311	–	–	I	F	–	–	–	–	–	–	–	M	–	–	–	V	N	D	–
	99–1327	–	–	I	F	–	–	–	–	–	–	–	M	–	–	–	V	N	D	–
	99–1338	–	–	I	F	–	–	–	–	–	–	–	M	–	–	–	V	N	D	–
	99–1320	–	–	I	F	–	–	–	–	–	–	–	M	–	–	–	V	N	D	–
	99–1329	–	–	I	F	–	–	–	–	–	–	–	M	–	–	–	V	N	D	–
	99–1328	–	–	I	F	–	–	–	–	–	–	–	M	–	–	–	V	N	D	–
	99–1333	–	–	I	F	–	–	–	–	–	–	–	M	–	–	–	V	N	D	–
	99–1312	–	–	I	–	–	–	V	–	–	–	A	M	N	–	–	V	N	D	–
	99–1316	–	–	I	–	–	–	V	–	–	–	A	M	–	–	–	V	N	D	–
	99–1323	–	–	I	–	–	–	V	–	–	–	A	M	–	–	–	V	N	D	–
	99–1335	–	–	I	–	–	–	V	–	–	–	A	M	N	–	–	V	N	D	–
	99–1315	–	V	I	–	G	T	–	T	P	N	G	–	–	D	N	V	N	D	–
	99–1336	–	V	I	–	G	T	–	T	P	N	G	–	–	D	N	V	N	D	–
	99–1310	–	V	I	–	–	–	–	–	–	–	–	–	N	–	–	–	N	D	–
	99–1319	–	V	I	–	–	–	–	–	–	–	–	–	–	–	–	–	N	D	–
	99–1314	–	V	I	–	–	–	–	–	–	–	–	–	–	–	–	–	N	D	–
	99–1332	–	V	I	–	–	–	–	–	–	–	–	–	–	–	–	–	N	D	–
	99–1324	–	V	I	–	–	–	–	–	–	–	–	–	–	–	–	–	N	D	–
	99–1330	F	–	–	F	–	–	–	–	–	–	–	M	–	–	–	–	–	–	–
	99–1334	–	–	–	–	–	–	–	–	–	–	–	–	–	–	–	–	–	–	–
	99–1339	–	–	–	–	–	–	–	–	–	–	–	–	–	–	–	–	–	–	–
	99–1318	–	–	–	–	–	–	–	–	–	–	–	–	–	–	–	–	–	–	–

		Positions					
		19	67	194	267	274	467
HPV-31	P17388|VL1*	S	S	S	T	T	R
	98–902	–	–	–	–	–	–
	98–751	–	–	–	–	–	–
	98–401	–	–	–	–	–	–
	98–299	T	–	–	–	–	G
	98–120	T	–	–	–	–	G
	99–1510	T	–	–	–	–	–
	99–1514	–	–	–	–	–	–
	99–1526	–	–	–	–	–	–
	99–1521	–	–	–	–	–	–
	99–1525	–	–	–	–	–	–
	99–1512	–	–	–	–	–	–
	99–1523	–	–	–	–	–	–
	99–1520	–	–	–	–	–	–
	99–1515	–	–	–	A	N	–
	99–1511	–	–	–	A	N	–
	99–1528	–	–	–	A	N	–
	99–1524	–	L	–	A	N	–
	99–1513	–	L	–	A	N	–
	99–1522	–	–	–	–	N	–
	99–1518	–	–	T	–	N	–
	99–1527	–	–	–	–	N	–

		Positions			
		56	133	266	385
HPV-33	P06416|VL1*	T	G	T	E
	98–274	N	S	K	D
	98–871	–	–	–	–
	99–1217	–	–	K	–
	99–1216	N	S	K	D
	99–1215	N	S	K	–
	99–1214	–	–	K	–
	99–1213	–	–	K	–
	99–1212	–	–	–	–
	99–1211	N	S	K	D
	99–1210	N	S	K	–

*UniProt accession numbers P26535, P17388, and P06416 were used as references for HPV genotypes 58, 31, and 33, respectively

#### HPV-33

Compared to the reference gene, the L1 sequence mutations of HPV-33 isolates showed nine variations (Additional file 1: Table S2). Among these, C797A, A1071G, and three changes C167A, G397A, and T885G occurred in 80.0% (n = 8), 70% (n = 7), and 50.0% (n = 5) of isolates, respectively. Table 2 represents all mutations in the amino acid sequences in comparison to the reference. Three replacements T266K, T56N, and G133S accounted for the most frequent amino acid changes.

#### HPV-58

Fourty seven nucleotides and 19 nonsynonymous mutations were observed in the L1 gene of HPV-58 isolates with 86.7% (n = 26) of each G430A, G1258A, C1263A, and A1264G, as well as G840A 83.3% (n = 25) as major substitutions (Additional file 1: Table S3). Among these variations, G430A, G1258A, and A1264G led to missense variations V144I, D420N, and N422D, respectively as predominant amino acid changes. Other variations are represented in Table 2.

### N-glycosylation analysis

N- and O-linked glycosylation are two main glycosylation in which glycans attach to side chains of Asparagine and mainly Serin/Threonine, respectively. N-glycosylation is a post translational modification that is involved in myriad biological process, such as protein folding, stability, and host cell membrane-ligand interactions [15]. Compared with the references, none of mutations in studied sequences led to any changes in N-glycosylation sites (Table 3). Although no changes were found between the N-glycosylated sites in references and HPV-58 and -31 isolates, at position 54 of HPV-33 reference a proline locates just after asparagine, which is predicted as a N-glycosylation site (Table 3). Since, in most cases, a proline situated after asparagine may inhibit the N-glycosylation by rendering the asparagine inaccessible, more experimental evidence is needed to confirm this N-link glycosylation site. In HPV-33 isolates 99–1217, 99–1216, 99–1215, 99–1211, 99–1210, and 98–274, threonine was replaced by asparagine at position 56 which was the only difference between these isolates and the N-glycosylation site in the reference.

**Table 3:** Prediction of N-glycosylation sites

Seq. name	Position	Potential*	N-Glycosylation result**
P26535 (HPV-58)	242 NKSD	0.6964	++
	252 NSTC	0.4706	−
	367 NMTL	0.5768	+
P06416 (HPV-33)	54 NPTN†	0.6644	++
	216 NKSD	0.6962	++
	341 NMTL	0.5742	+
P17388 (HPV-31)	263 NRSG	0.5888	+
	289 NSTY	0.6082	+
	342 NMSV	0.5354	+

*The potential scores indicate the averaged output of neural networks

**Within the protein sequences, the position with possible crossing the threshold of 0.5 is predicted N-glycosylated, while the positions with below the cutoff are predicted as negative sites

†N-link glycosylated asparagine within Asn-Pro-Ser/Thr sequon

### Selective analysis

During natural selection some variants can be adapted or deleted in a population. In positive selection, the variants that confer a fitness advantage are fixed in the population, while in negative selection the variants with a deleterious effect on the fitness are gradually removed [16]. Datamonkey server estimates alpha and beta substitution rates codon-by-codon under selection. A nonsynonymous replacement with a negligible effect on its fitness is classifies as neutral but a variation which leads to an increase in fitness, is considered a positive selection. Also, a purifying substitution is interpreted as a negative selection that means the mutation may gradually be removed from the genome. Tables 4, 5 and 6 indicates the codon-by-codon results from the FEL analysis in case of HPV genotypes 31, 33, and 58. Among 85 nucleotide variations which were under different selective pressures, 43.5% (n = 37) were under negative selection and 55.3% (n = 47) accounted for neutral selection. 17, 17, and 3 codons with negative (purifying) selection were observed in the HPV-58, -31, and -33 sequences, respectively. Only one positive (diversifying) selection at position 150 in the HPV-58 sequences was found. We reported all neutral variations in the case of these HPV genotypes in Additional file 1: Table S4a–c.

**Table 4:** The codon-by-codon results from the FEL analysis for HPV-58 isolates

Codon	Alpha	Beta	Alpha = beta	*p*-value	Class
19	14.324	0	1.91	0.045	Purifying selection
75	51.571	0	4.29	0.0331	
79	54.106	0	8.597	0.0002	
85	15.701	0	2.213	0.0436	
99	16.633	0	5.808	0.0328	
152	29.192	0	4.17	0.0074	
158	14.732	0	4.153	0.0247	
217	56.116	0	7.702	0.0586	
280	27.852	0	5.124	0.015	
292	39.978	0	8.626	0.0322	
298	14.928	0	4.618	0.0313	
312	16.334	0	5.264	0.0345	
385	56.116	0	7.702	0.0586	
455	12.283	0	2.011	0.0647	
482	8.67	0	2.099	0.0994	
484	22.008	0	9.996	0.0374	
505	38.551	0	17.189	0.0066	
150	0	20.849	9.975	0.0818	Diversifying selection

**Table 5:** The codon-by-codon results from the FEL analysis for HPV-33 isolates

Codon	Alpha	Beta	Alpha = beta	*p*-value	Class
272	49.148	0	12.747	0.0239	Purifying selection
295	57.03	0	7.626	0.0472	
357	77.23	0	12.207	0.0574

**Table 6:** The codon-by-codon results from the FEL analysis for HPV-31 isolates

Codon	Alpha	Beta	Alpha = beta	*p*-value	Class
10	25.466	0	3.258	0.0478	Purifying selection
17	12.981	0	2.651	0.0798	
59	31.621	0	3.314	0.0476	
156	15.197	0	3.121	0.0781	
178	17.723	0	2.872	0.053	
187	23.489	0	2	0.0411	
192	21.71	0	3.305	0.0537	
229	43.157	0	4.242	0.0348	
259	8.696	0	2.112	0.0934	
272	34.2	0	6.988	0.0112	
339	21.754	0	2.412	0.0370	
345	15.762	0	3.432	0.0756	
422	13.943	0	3.233	0.0927	
430	111.607	0	6.825	0.004	
446	15.619	0	3.284	0.0854	
489	13.703	0	4.546	0.0361	
500	73.185	0	6.658	0.0049	

Alpha, Synonymous substitution rate at a site; beta, non-synonymous substitution rate at a site; alpha = beta, the rate estimate under the neutral model

### Homology analysis on L1 loops

**Fig. 2:**
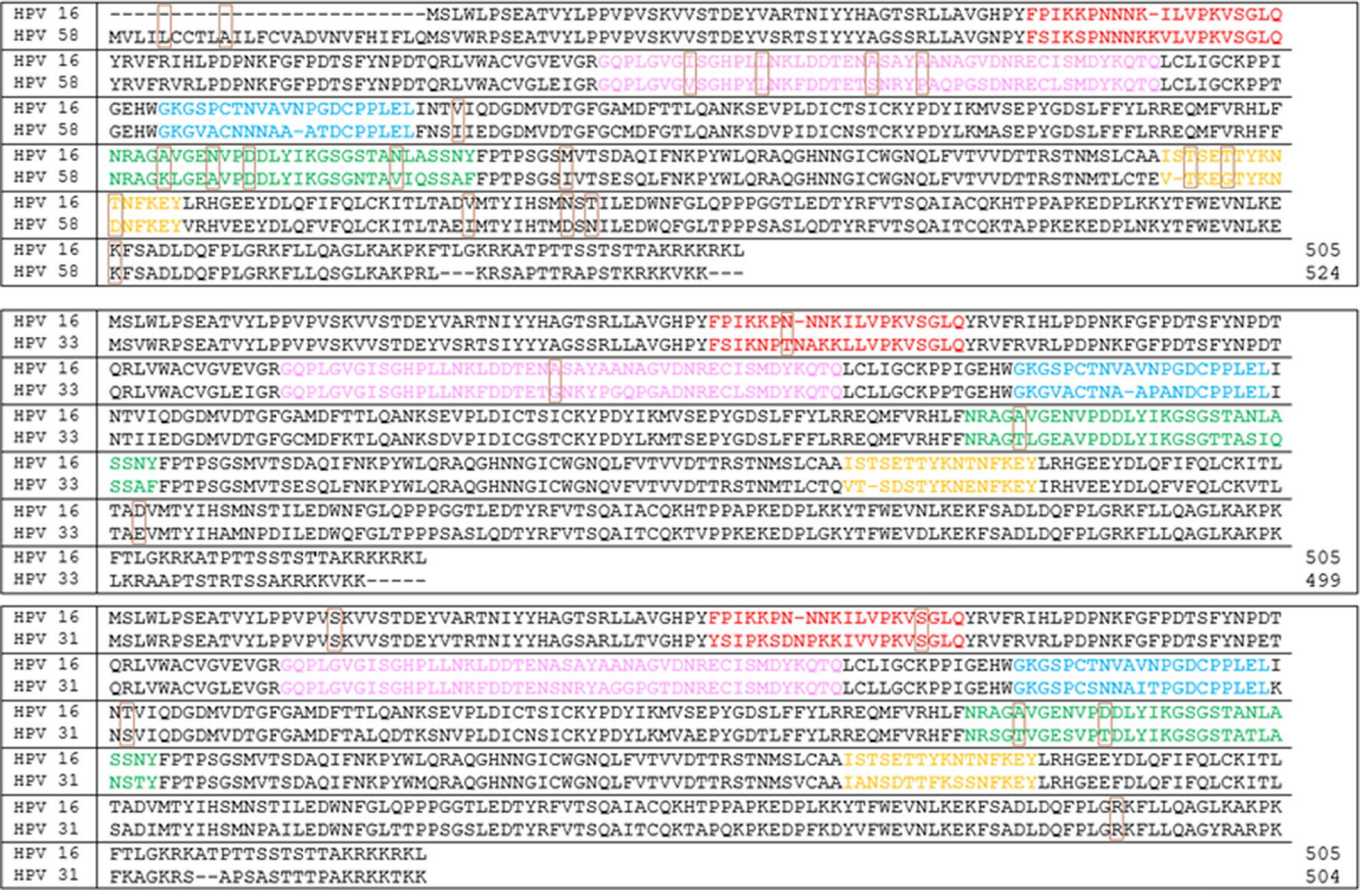
The L1 gene in reference genomes of HPV-58, -33, and -31 compared with that of HPV-16. Red = BC Loop, pink = DE Loop, blue = EF Loop, green = FG Loop, orange = HI Loop. The mutations in the understudied isolates are presented by brown boxes

According to the HPV**-**16 major capsid protein, the pentameric L1 protein consists of five external surface loops, which are designated as BC, DE, EF, FG, and HI loop [17]. The structure of the HPV**-**16 L1 protein was compared with each HPV-31, HPV-33, and HPV-58 references by using homology model to predict the changes in the L1 loops (Fig. 2). Among missense variations in HPV-31 samples, T267A and T274N were in FG loop and S67L in BC loop. Out of four nonsynonymous replacements in the HPV-33 isolates, three changes T56N, G133S, and T266K were located in BC, DE, and FG loop, respectively. Among nineteen changes in the HPV-58 amino acid sequences, eleven replacements were located in DE, FG, and HI loops, including V144I, L150F, S159G, and P163T in the DE loop; K292T, A296P, D299N, and V311A/G in the FG loop; T375N, G378D, and D383N in the HI loop. Remaining mutations occurred out of the loops.

## Discussion

As HPV-16 and -18 comprise 70% of worldwide cervical cancers [3], in Iran like other parts of the world, most research has been conducted of these two genotypes, focusing on their prevalence, genetic variability, carcinogenicity, integration status, and lineages [18–21]. According to our previous study, HPV-52 and -58 followed by HPV-31 were the second more common types of the virus after HPV-16 [22], which indicates the importance of other HR HPVs, including HPV-31, -33, and -58. Furthermore, the L1 gene can be used for (sub)lineage classification and is critical for the induction of neutralizing antibodies [23]. Therefore, we decided to investigate the amino acid changes and genetic diversity of the L1 gene and attempted to fill the gap as the information about HPV-31, -33, and -58 in Iran is extensively restricted.

Our phylogenetic analysis illustrated that 63.3% of HPV-58 isolates fell into the lineage B, 16.7% in D, 13.3% in A, and 6.7% in C lineages. The lineage A accounted for 100.0% of HPV-33 samples. Among HPV-31 samples, 61.9%, 23.8%, and 14.3% were in lineages A, C, and B, respectively. Our findings about HPV-31 are in consistent with Hosseini’s findings that variants A and C are common in Iran [24] but to the best of our knowledge, this study is the first in Iran. Some variants of HPV-16 are more aggressive than others and even lineage-specific nucleotide changes were reported [25, 26]. Our samples were collected from females with normal pap-test; however, as shown by previous studies the (sub)lineage of other HR HPVs, like HPV-16, can be involved in the development of neoplasia. On the other hand, few studies were undertaken on the genetic variability in the L1 gene of HPV genotypes 31, 33, and 58 to identify the nucleotide changes associated with cervical cancer and more studies focused on E6 and E7 regions. For example, the association of HPV-31, -33, and -58 variants with the risk of high grade of abnormal lesions was reported. Women with A and B variants were at greater risk of HPV-31-related severe lesions [27]. Sub-lineage A1 in HPV-33 has been mostly relevant to cervical cancer [5]; however, due to the low number of understudied samples more studies are required to confirm this conclusion. Also, the A variants of HPV-58 was found to be associated with perseverance and severity of lesions [28, 29]. In addition to oncogenicity, nucleotide changes in L1 may have critical role in protein folding and self-assembly process [30], and even sensitivity to neutralizing antibodies (HPV-33).

Negative selection or purifying selection focuses on deleterious mutations [16]. In contrast, variations with positive pressure contribute to productive infection. Host factors, including immune cells, and viral proteins also influence positive selection [31] and can even be a main factor in HPV persistency. We only found one codon position in HPV-58 sequences that is under positive selection, at position 150, and no negative selection site was observed. N-glycosylation highly affects the protein stability and its third structure, especially in interaction with another protein and receptor signaling, which this modification has been mainly conserved in evolution [32]. As it was expected, no difference was observed between our sequences and those of the references.

The majority of differences among HPV-16 and other HPV genotypes are observed in the five surface-exposed loops and C-termini, against which anti bodies are produced. A comparison of the L1 amino acid sequences with the HPV-16 L1 reveals the main homology in internal regions of the L1 capsid. 41.4% (12/29) of nonsynonymous mutations of our samples occurred out of the loops and among those mutations occurring in loops (58.6%, 17/29) they were mostly located in DE and FG loops (70.6%). The FG and DE loops of L1 proteins contribute to the epitopes for cross-neutralizing antibodies. Deletion at positions 281 and 282, which are located in the late region of the FG loop, leads to the loss of cross-neutralizing antibodies, which are produced through vaccines containing HPV-16 virus-like particles (VLPs) [9]. In this study, although 70.6% (n = 12) missense mutations were located in the FG and DE loops, none of them occurred at positions 281–282. Furthermore, sub-lineages can influence the intensity of the immune responses. Godi and colleagues illustrated that HPV-33 A1 variants were more sensitive than other HPV-33 sub-lineages against the cross-neutralizing antibodies produced by 4-valent vaccine. They pointed that the occurrence of amino acid changes in the FG and DE loops of A2, B, and C (sub)lineages was the main cause for the difference in antibody responses [33]. More studies are needed to find the impact of these mutations in both FG and DE loops on the neutralizing antibodies.

This study had some limitations which should be addressed; (1) According to the report of Ministry of Health of Iran, cervical cancer did not rank in ten common cancers among Iranian women. As it was highly unlike to collect adequate positive cancerous samples for HPV-31, -33, and -58, we designed our study on samples from women with normal cytology to estimate the predominant circulating genotypes of interest in the population. However, our sample size of positive specimens for the three HPV genotypes was small. (2) Our study contained only samples with normal cytology. In contrast, some specific-lineage variations in coding sequence regions or the long control region (LCR) of HPV-16 are associated with severe lesions [25]. Thus, investigations on other regions and samples with high grade neoplasia are needed. (3) The region/gene that we selected may have an effect on sub-lineage classification. The accuracy of L1 in terms of lineage and sub-lineage classification is (97.75 and 97.75) and (100 and 48.72) for HPV-58 and -16, respectively [34], which proves L1 to be an appropriate gene for HPV-58 classification, while E2 was reported the best region for sub-lineage HPV-16 classification. Although variations in one gene is compatible with changes in HPV complete genome, different regions have different accuracy in lineage and sub-lineage classification.; therefore, more studies on different genes/regions, especially for HPV-31 and -33 is suggested to find the best region for sub-lineage classification.

## Conclusion

In the current study, phylogenetic analysis of HPV-58, -33, and -31 showed B1, A, and A1 as the frequent (sub)lineages circulating among women with normal cytology. We also reported, for the first time, the genetic diversity in the L1 loops of these HPV genotypes and the effect of amino acid changes on selective pressure and N-glycosylation sites. Additional studies with larger sample size, containing cancerous samples, in different geographical regions in Iran are required to provide a reliable insight into the distribution of these HR HPV genotypes.

## Supplementary Information

**Additional file 1.** Single nucleotide mutations and neutral variations in the understudied HPV genotypes 31, 33, and 58 isolates.

## Data Availability

The data supporting the findings of this study are available in GenBank, both the current study, and the cited references.
