# Supplementary material for: Genetic diversity and bioinformatic analysis in the L1 gene of HPV genotypes 31, 33, and 58 circulating in women with normal cervical cytology
Source: Infect Agent Cancer. 2023 Mar 23;18:19. doi: 10.1186/s13027-023-00499-7 (PMC10037780; doi:10.1186/s13027-023-00499-7)
Supplement: Supplementary file 1 — Additional file 1. Single nucleotide mutations and neutral variations in the understudied HPV genotypes 31, 33, and 58 isolates. [file 13027_2023_499_MOESM1_ESM.docx]

**Supplementary Table 1.** Single nucleotide mutations in HPV-31 isolates.

|  | Isolate | 30 | 51 | 55 | 177 | 200 | 294 | 297 | 369 | 370 | 468 | 534 | 561 | 576 | 580 | 654 | 687 | 777 | 799 | 816 | 821 | 828 | 1017 |
| --- | --- | --- | --- | --- | --- | --- | --- | --- | --- | --- | --- | --- | --- | --- | --- | --- | --- | --- | --- | --- | --- | --- | --- |
| HPV-31 Ref * | | T | A | T | A | C | C | A | A | T | A | C | T | A | T | A | T | G | A | C | C | A | T |
| A1 | 98-902 | - | - | - | - | - | - | - | - | - | ­- | - | - | - | - | - | - | - | - | - | - | - | - |
| A1 | 98-751 | - | - | - | - | - | - | - | - | - | - | - | - | - | - | - | - | - | - | - | - | - | - |
| A1 | 98-401 | - | - | - | - | - | - | - | - | - | - | - | - | - | - | - | - | - | - | - | - | - | - |
| A1 | 98-299 | - | - | A | - | - | - | - | - | - | - | - | - | - | - | - | - | - | - | - | - | - | - |
| A1 | 98-120 | - | - | A | - | - | - | - | - | - | - | - | - | - | - | - | - | - | - | - | - | - | - |
| A1 | 99-1510 | - | - | A | - | - | - | - | - | - | - | - | - | - | - | - | - | - | - | - | - | - | - |
| A1 | 99-1514 | - | G | - | - | - | - | - | - | - | - | - | - | - | - | - | - | - | - | - | - | - | - |
| A1 | 99-1526 | - | - | - | - | - | - | - | - | - | - | - | - | - | - | - | - | - | - | - | - | - | - |
| A1 | 99-1521 | - | - | - | - | - | - | - | - | - | - | - | - | - | - | - | - | - | - | T | - | - | - |
| A1 | 99-1525 | - | - | - | - | - | - | - | - | - | - | - | - | - | - | - | - | - | - | T | - | - | - |
| A1 | 99-1512 | - | G | - | - | - | - | - | - | - | - | - | - | - | - | - | - | - | - | T | - | - | - |
| A1 | 99-1523 | - | - | - | - | - | - | - | - | - | - | - | - | - | - | - | - | - | - | T | - | - | - |
| A2 | 99-1520 | - | - | - | - | - | - | - | - | - | - | - | - | - | - | - | - | - | - | T | - | - | - |
| C3 | 99-1515 | A | - | - | - | - | - | - | G | C | G | T | C | - | - | - | - | A | G | T | A | - | - |
| C3 | 99-1511 | A | - | - | - | - | - | - | G | C | G | T | C | - | - | - | - | A | G | T | A | - | - |
| C3 | 99-1528 | A | - | - | G | - | - | - | G | C | G | T | C | - | - | - | - | A | G | T | A | - | - |
| C1 | 99-1524 | A | - | - | - | T | T | - | - | C | G | T | - | - | - | - | - | A | G | T | A | - | - |
| C1 | 99-1513 | A | - | - | - | T | - | - | - | C | G | T | - | - | - | - | - | A | G | T | A | - | - |
| B2 | 99-1522 | - | - | - | - | - | - | G | - | C | - | - | - | G | - | G | A | - | - | T | A | G | C |
| B1 | 99-1518 | - | - | - | - | - | - | - | - | C | - | - | - | G | A | - | A | - | - | T | A | - | - |
| B2 | 99-1527 | - | - | - | - | - | - | G | - | C | - | - | - | G | - | - | A | - | - | T | A | G | C |

**Supplementary Table 1. Continued**

|  | Isolate | 1035 | 1152 | 1221 | 1245 | 1266 | 1290 | 1311 | 1338 | 1399 | 1401 | 1467 | 1500 |
| --- | --- | --- | --- | --- | --- | --- | --- | --- | --- | --- | --- | --- | --- |
| HPV-31 Ref* | | T | A | G | G | C | A | C | A | C | C | C | A |
|  | 98-902 | - | - | - | - | A | - | - | - | - | - | - | - |
|  | 98-751 | - | - | - | - | A | - | - | - | - | - | - | - |
|  | 98-401 | - | - | - | - | A | - | - | - | - | - | - | - |
|  | 98-299 | - | - | - | - | A | - | - | - | G | - | G | - |
|  | 98-120 | - | - | - | - | A | G | - | - | G | - | G | - |
|  | 99-1510 | - | - | - | - | A | G | - | - | - | - | - | - |
|  | 99-1514 | - | - | - | - | A | - | - | - | - | - | - | - |
|  | 99-1526 | - | - | - | - | A | - | - | - | - | - | - | - |
|  | 99-1521 | - | - | - | - | A | - | - | - | - | - | - | - |
|  | 99-1525 | - | - | - | - | A | - | - | - | - | - | - | - |
|  | 99-1512 | - | - | - | - | A | - | - | - | - | - | - | - |
|  | 99-1523 | - | - | - | - | A | - | - | - | - | - | G | - |
|  | 99-1520 | - | - | - | - | A | - | - | - | - | - | - | - |
|  | 99-1515 | G | - | - | A | A | - | - | - | - | - | - | G |
|  | 99-1511 | G | - | - | A | A | - | - | - | - | T | - | - |
|  | 99-1528 | G | - | - | A | A | - | - | - | - | - | - | - |
|  | 99-1524 | G | G | - | A | A | - | - | C | - | - | - | - |
|  | 99-1513 | G | - | - | A | A | - | - | C | - | - | - | - |
|  | 99-1522 | - | - | A | A | A | G | T | - | - | - | - | G |
|  | 99-1518 | - | - | A | A | A | - | - | - | - | - | - | - |
|  | 99-1527 | - | - | A | A | A | - | T | - | - | - | - | G |

The positions of nucleotide changes are written vertically across the top. The absence of mutations relative to the reference is represented by dashes.

* HPV-31 Ref= J04353.1

**Supplementary Table 2.** Single nucleotide mutations in HPV-33 isolates.

|  | Isolate | 167 | 397 | 797 | 816 | 885 | 999 | 1071 | 1155 | 1387 |
| --- | --- | --- | --- | --- | --- | --- | --- | --- | --- | --- |
| HPV-33 Ref * |  | C | G | C | C | T | G | A | A | T |
| A1 | 98-871 | - | - | - | G | - | - | - | - | C |
| A1 | 99-1212 | - | - | - | - | - | - | G | - | - |
| A1 | 99-1213 | - | - | A | - | - | - | - | - | C |
| A1 | 99-1214 | - | - | A | G | - | - | G | - | - |
| A1 | 99-1217 | - | - | A | - | - | - | - | - | C |
| A2 | 98-274 | A | A | A | - | C | - | G | C | - |
| A2 | 99-1216 | A | A | A | - | C | - | G | C | - |
| A2 | 99-1210 | A | A | A | - | C | A | G | - | - |
| A2 | 99-1211 | A | A | A | - | C | - | G | C | - |
| A2 | 99-1215 | A | A | A | - | C | - | G | - | - |

The positions of nucleotide changes are written vertically across the top. The absence of mutations relative to the reference is represented by dashes.

*HPV-33 Ref= M12732.1

**Supplementary Table 3.** Single nucleotide mutations in HPV-58 isolates.

|  | Isolate | 15 | 29 | 57 | 102 | 225 | 237 | 243 | 255 | 297 | 375 | 408 | 430 | 450 | 456 | 474 | 475 | 487 | 520 | 642 | 651 | 658 | 840 | 875 | 876 | 886 |
| --- | --- | --- | --- | --- | --- | --- | --- | --- | --- | --- | --- | --- | --- | --- | --- | --- | --- | --- | --- | --- | --- | --- | --- | --- | --- | --- |
| HPV-58 Ref* | | A | C | C | G | T | A | C | A | C | G | G | G | A | A | C | A | G | T | G | T | A | G | A | A | G |
| B1 | 13 | - | - | - | A | - | G | - | - | - | - | A | A | C | G | - | - | - | - | - | - | - | A | - | G | - |
| B1 | 98-368 | - | - | - | A | - | G | - | - | - | - | A | A | C | G | - | - | - | - | - | - | - | A | - | G | - |
| B1 | 98-201 | - | - | - | A | - | G | - | - | - | - | A | A | C | G | - | - | - | - | - | - | - | A | - | G | - |
| B1 | 98-453 | - | - | - | A | - | G | - | - | - | - | A | A | C | G | - | - | - | - | - | - | - | A | - | G | - |
| B1 | 98-975 | - | - | - | A | - | G | - | - | - | - | A | A | C | G | - | - | - | - | - | - | - | A | - | G | - |
| B1 | 98-799 | - | - | - | A | - | G | - | - | - | - | A | A | C | G | - | - | - | - | - | - | - | A | - | G | - |
| B1 | 98-723 | - | - | - | A | - | G | - | - | - | - | A | A | C | G | - | - | - | - | - | - | - | A | - | G | - |
| B1 | 98-611 | - | - | - | A | - | G | - | - | - | - | A | A | C | G | - | - | - | - | - | - | - | A | - | G | - |
| B1 | 99-1311 | - | - | - | A | - | G | - | - | - | - | A | A | C | G | - | - | - | - | - | - | - | - | - | G | - |
| B1 | 99-1327 | - | - | - | A | - | G | T | - | - | - | A | A | C | G | - | - | - | - | - | - | - | A | - | G | - |
| B1 | 99-1338 | - | - | - | A | - | G | - | - | - | - | A | A | C | G | - | - | - | - | - | - | - | A | - | G | - |
| B1 | 99-1320 | - | - | - | A | - | G | - | - | - | - | A | A | C | G | - | - | - | - | - | - | - | A | - | G | - |
| B1 | 99-1329 | - | - | - | A | - | G | - | - | - | - | A | A | C | G | - | - | - | - | - | - | - | A | - | G | - |
| B1 | 99-1328 | - | - | - | A | - | G | - | - | - | - | A | A | C | G | - | - | - | - | - | - | - | A | - | G | - |
| B1 | 99-1333 | - | - | T | A | - | G | - | - | - | - | A | A | C | G | - | - | - | - | - | - | - | A | - | G | - |
| B2 | 99-1312 | - | - | - | - | C | G | - | - | - | - | - | A | - | - | - | - | - | - | A | - | G | A | - | - | - |
| B2 | 99-1316 | - | - | - | - | C | G | - | - | - | - | - | A | - | - | - | - | - | C | - | - | G | A | - | - | - |
| B2 | 99-1323 | - | - | - | - | C | G | - | - | - | - | - | A | - | - | - | - | - | - | - | - | G | A | - | - | - |
| B2 | 99-1335 | - | - | - | - | C | G | - | - | - | - | - | A | - | - | - | - | - | - | - | - | G | A | - | - | - |
| C | 99-1315 | - | T | - | - | - | G | - | - | G | A | - | A | - | - | T | G | A | - | - | - | - | A | C | C | C |
| C | 99-1336 | - | T | - | - | - | - | - | - | G | A | - | A | - | - | T | G | A | - | - | - | - | A | C | C | C |
| D | 99-1310 | - | T | - | - | - | - | - | G | - | - | - | A | - | - | - | - | - | - | - | - | - | A | - | - | - |
| D | 99-1319 | - | T | - | - | - | - | - | G | - | - | - | A | - | - | - | - | - | - | - | - | - | A | - | - | - |
| D | 99-1314 | - | T | - | - | - | - | - | G | T | - | - | A | - | - | - | - | - | - | - | - | - | A | - | - | - |
| D | 99-1332 | - | T | - | - | - | - | - | G | - | - | - | A | - | - | T | - | - | - | - | - | - | A | - | - | - |
| D | 99-1324 | - | T | - | - | - | - | - | G | - | - | - | A | - | - | - | - | - | - | - | - | - | A | - | G | - |
| A3 | 99-1330 | C | - | - | - | - | - | - | - | - | - | - | - | C | G | - | - | - | - | - | - | - | - | - | - | - |
| A1 | 99-1334 | - | - | - | - | - | - | - | - | - | - | - | - | - | - | - | - | - | - | - | C | - | - | - | - | - |
| A1 | 99-1339 | - | - | - | - | - | - | - | - | - | - | - | - | - | - | - | - | - | - | - | - | - | - | - | - | - |
| A1 | 99-1318 | - | - | - | - | - | G | - | - | - | - | - | - | - | - | - | - | - | - | - | - | - | - | - | - | - |

**Supplementary Table 3. Continued**

| Isolate | 894 | 895 | 932 | 936 | 975 | 1124 | 1125 | 1128 | 1133 | 1147 | 1155 | 1234 | 1258 | 1263 | 1264 | 1365 | 1436 | 1446 | 1450 | 1452 | 1515 | 1524 |
| --- | --- | --- | --- | --- | --- | --- | --- | --- | --- | --- | --- | --- | --- | --- | --- | --- | --- | --- | --- | --- | --- | --- |
| HPV-58 Ref * | G | G | T | C | A | C | T | G | G | G | T | A | G | C | A | A | A | A | C | A | A | G |
| 13 | - | - | - | - | G | - | - | - | - | - | - | G | A | A | G | - | - | - | - | - | G | A |
| 98-368 | - | - | - | - | G | - | - | - | - | - | - | G | A | A | G | - | - | - | T | - | G | A |
| 98-201 | - | - | - | - | G | - | - | - | - | - | - | G | A | A | G | - | - | - | T | - | G | A |
| 98-453 | - | - | - | - | G | - | - | - | - | - | - | G | A | A | G | - | - | - | - | - | G | A |
| 98-975 | - | - | - | - | G | - | - | - | - | - | - | G | A | A | G | - | C | - | T | - | G | A |
| 98-799 | - | - | - | - | G | - | - | - | - | - | - | G | A | A | G | - | - | - | T | - | G | A |
| 98-723 | - | - | - | - | G | - | - | - | - | - | - | G | A | A | G | - | - | - | T | - | G | A |
| 98-611 | - | - | - | - | G | - | - | - | - | - | - | G | A | A | G | - | - | - | - | - | G | A |
| 99-1311 | - | - | - | - | G | - | - | - | - | - | - | G | A | A | G | - | - | - | - | - | G | A |
| 99-1327 | - | - | - | - | G | - | - | - | - | - | - | G | A | A | G | - | - | - | - | - | G | A |
| 99-1338 | - | - | - | - | G | - | - | - | - | - | - | G | A | A | G | - | - | - | - | - | G | A |
| 99-1320 | - | - | - | - | G | - | - | - | - | - | - | G | A | A | G | - | - | - | - | - | G | A |
| 99-1329 | - | - | - | - | G | - | - | - | - | - | - | G | A | A | G | - | - | - | - | - | G | A |
| 99-1328 | - | - | - | - | G | - | - | - | - | - | - | G | A | A | G | - | - | - | - | - | G | A |
| 99-1333 | - | - | - | - | G | - | - | - | - | - | - | G | A | A | G | - | - | - | - | - | G | A |
| 99-1312 | T | - | C | T | G | A | - | - | - | - | - | G | A | A | G | - | - | - | - | - | G | - |
| 99-1316 | T | - | C | T | G | - | - | - | - | - | - | G | A | A | G | - | - | - | - | - | - | - |
| 99-1323 | - | - | C | T | G | - | - | - | - | - | - | G | A | A | G | - | - | - | - | - | - | - |
| 99-1335 | T | - | C | T | G | A | - | - | - | - | - | G | A | A | G | - | - | - | - | - | - | - |
| 99-1315 | A | A | G | - | - | - | - | A | A | A | - | G | A | A | G | - | - | - | - | T | - | A |
| 99-1336 | A | A | G | - | - | - | - | A | A | A | - | G | A | A | G | - | - | - | - | T | - | A |
| 99-1310 | A | - | - | - | - | A | - | - | - | - | - | - | A | A | G | - | - | G | - | G | C | - |
| 99-1319 | A | - | - | - | - | - | - | - | - | - | - | - | A | A | G | - | - | G | - | G | C | - |
| 99-1314 | A | - | - | - | - | - | - | - | - | - | - | - | A | A | G | - | - | - | - | G | C | - |
| 99-1332 | A | - | - | - | - | - | - | - | - | - | - | - | A | A | G | - | - | G | - | G | - | - |
| 99-1324 | A | - | - | - | - | - | - | - | - | - | - | - | A | A | G | - | - | G | - | G | C | - |
| 99-1330 | - | - | - | T | G | - | - | - | - | - | - | - | - | - | - | - | - | - | - | - | - | - |
| 99-1334 | - | - | - | - | - | - | - | - | - | - | - | - | - | - | - | G | - | - | - | - | - | - |
| 99-1339 | - | - | - | - | - | - | C | - | - | - | - | - | - | - | - | - | - | - | - | - | - | - |
| 99-1318 | - | - | - | - | - | - | - | - | - | - | C | - | - | - | - | - | - | - | - | - | - | - |

The positions of nucleotide changes are written vertically across the top. The absence of mutations relative to the reference is represented by dashes.

*HPV-58 Ref =D90400.

**Supplementary Table 4a.** All site-by-site neutral selection of HPV-31 isolates.

| Codon | Alpha | Beta | alpha=beta | p-value | Class |
| --- | --- | --- | --- | --- | --- |
| 19 | 0 | 5.297 | 4.336 | 0.5324 | Neutral  Selection |
| 67 | 0 | 11.626 | 6.075 | 0.2533 |  |
| 98 | 6.84 | 0 | 1.945 | 0.1128 |  |
| 99 | 13.486 | 0 | 5.554 | 0.1988 |  |
| 123 | 15.669 | 0 | 6.045 | 0.1693 |  |
| 124 | 9.292 | 0 | 3.825 | 0.1855 |  |
| 194 | 0 | 11.584 | 6.022 | 0.2542 |  |
| 267 | 0 | 3.756 | 2.236 | 0.31 |  |
| 274 | 0 | 4.184 | 3.775 | 0.6583 |  |
| 276 | 13.486 | 0 | 5.554 | 0.1988 |  |
| 384 | 14.07 | 0 | 5.696 | 0.1923 |  |
| 407 | 8.127 | 0 | 4.147 | 0.2514 |  |
| 415 | 12.283 | 0 | 5.407 | 0.1950 |  |
| 437 | 7.292 | 0 | 2.314 | 0.1305 |  |
| 467 | 6.765 | 2.739 | 3.914 | 0.5324 |  |

**Supplementary Table 4b.** All site-by-site neutral selection of HPV-33 isolates.

| Codon | Alpha | Beta | alpha=beta | p-value | Class |
| --- | --- | --- | --- | --- | --- |
| 56 | 0.644 | 13.385 | 13.073 | 0.7793 | Neutral  Selection |
| 133 | 0.349 | 15.991 | 15.139 | 0.7598 |  |
| 266 | 0 | 33.692 | 28.428 | 0.4245 |  |
| 333 | 39.889 | 0 | 16.277 | 0.1836 |  |
| 385 | 0 | 14.453 | 13.164 | 0.6683 |  |
| 463 | 41.605 | 0 | 20.018 | 0.2335 |  |

**Supplementary Table 4c.** All site-by-site neutral selection of HPV-58 isolates.

| Codon | Alpha | Beta | alpha=beta | p-value | Class |
| --- | --- | --- | --- | --- | --- |
| 5 | 0 | 11.288 | 4.78 | 0.1903 | Neutral  Selection |
| 10 | 0 | 3.372 | 3.012 | 0.6359 |  |
| 34 | 6.426 | 0 | 1.849 | 0.1155 |  |
| 81 | 6.333 | 0 | 2.19 | 0.1464 |  |
| 125 | 5.416 | 0 | 3.481 | 0.3505 |  |
| 136 | 5.021 | 0 | 1.578 | 0.1303 |  |
| 144 | 0 | 3.165 | 2.672 | 0.5653 |  |
| 159 | 0 | 2.274 | 2.141 | 0.7334 |  |
| 163 | 0 | 3.362 | 2.177 | 0.3516 |  |
| 174 | 8.67 | 0 | 4.719 | 0.2806 |  |
| 214 | 5.232 | 0 | 3.355 | 0.3557 |  |
| 220 | 0 | 4.027 | 3.619 | 0.6574 |  |
| 296 | 0 | 3.618 | 3.182 | 0.6266 |  |
| 299 | 0 | 2.687 | 2.546 | 0.718 |  |
| 311 | 0 | 7.835 | 6.839 | 0.4757 |  |
| 325 | 0 | 5.886 | 5.589 | 0.6487 |  |
| 375 | 32.12 | 5.996 | 8.295 | 0.2411 |  |
| 376 | 6.426 | 0 | 1.787 | 0.1102 |  |
| 378 | 0 | 2.369 | 2.192 | 0.6946 |  |
| 383 | 0 | 2.687 | 2.546 | 0.718 |  |
| 412 | 0 | 9.21 | 6.806 | 0.233 |  |
| 420 | 0 | 2.724 | 2.56 | 0.7169 |  |
| 421 | 8.955 | 0 | 4.559 | 0.2631 |  |
| 422 | 0 | 2.743 | 2.569 | 0.7165 |  |
| 479 | 0 | 2.527 | 1.751 | 0.3972 |  |
| 508 | 11.504 | 0 | 7.162 | 0.1794 |  |

Alpha, Synonymous substitution rate at a site; beta, non-synonymous substitution rate at a site; alpha=beta, The rate estimate under the neutral mod
